# Different types of uncertainty distinguished by monkey prefrontal neurons

**DOI:** 10.1093/texcom/tgac002

**Published:** 2022-01-11

**Authors:** Madoka Matsumoto, Hiroshi Abe, Keiji Tanaka, Kenji Matsumoto

**Affiliations:** Department of Preventive Intervention for Psychiatric Disorders, National Institute of Mental Health, National Center of Neurology and Psychiatry, 4-1-1 Ogawahigashi, Kodaira, Tokyo 187-8553, Japan; Brain Science Institute, Tamagawa University, 6-1-1 Tamagawa-gakuen, Machida, Tokyo 194-8610, Japan; Laboratory for Molecular Analysis of Higher Brain Function, Center for Brain Science, RIKEN, 2-1 Hirosawa, Wako, Saitama 351-0198, Japan; Laboratory for Cognitive Brain Mapping, Center for Brain Science, RIKEN, 2-1 Hirosawa, Wako, Saitama 351-0198, Japan; Laboratory for Molecular Analysis of Higher Brain Function, Center for Brain Science, RIKEN, 2-1 Hirosawa, Wako, Saitama 351-0198, Japan; Laboratory for Cognitive Brain Mapping, Center for Brain Science, RIKEN, 2-1 Hirosawa, Wako, Saitama 351-0198, Japan; Laboratory for Cognitive Brain Mapping, Center for Brain Science, RIKEN, 2-1 Hirosawa, Wako, Saitama 351-0198, Japan; Brain Science Institute, Tamagawa University, 6-1-1 Tamagawa-gakuen, Machida, Tokyo 194-8610, Japan; Laboratory for Cognitive Brain Mapping, Center for Brain Science, RIKEN, 2-1 Hirosawa, Wako, Saitama 351-0198, Japan

**Keywords:** frontal lobe, medial prefrontal cortex, nonhuman primate, prediction error, single-unit recording

## Abstract

To adapt one’s behavior, in a timely manner, to an environment that changes in many different aspects, one must be sensitive to uncertainty about each aspect of the environment. Although the medial prefrontal cortex has been implicated in the representation and reduction of a variety of uncertainties, it is unknown whether different types of uncertainty are distinguished by distinct neuronal populations. To investigate how the prefrontal cortex distinguishes between different types of uncertainty, we recorded neuronal activities from the medial and lateral prefrontal cortices of monkeys performing a visual feedback-based action-learning task in which uncertainty of coming feedback and that of context change varied asynchronously. We found that the activities of two groups of prefrontal cells represented the two different types of uncertainty. These results suggest that different types of uncertainty are represented by distinct neural populations in the prefrontal cortex.

## Introduction

To live in an environment in which things are ever changing, we have to detect uncertainty in the environment and adapt our behaviors to make outcomes of our behaviors certain (Bach and Dolan 2012; Ma and Jazayeri 2014; Soltani and Izquierdo 2019; Koblinger et al. 2021). As the environment is complicated, there can be various types of uncertainty, each of which requires a distinct type of adaptation. Therefore, we must deal with different types of uncertainty differently. Bach and Dolan (Bach and Dolan 2012) integrated theoretical concepts of uncertainty into a decision-making framework and proposed a hierarchical processing model of uncertainty with four distinct (i.e. sensory, state, rule, and outcome) uncertainties. However, there is a debate about uncertainty representation in the brain, whether different types of uncertainty are represented in different neural networks or in a unified network (Bach and Dolan 2012; Ma and Jazayeri 2014; Koblinger et al. 2021). Some theories assume that uncertainty about the value of a particular variable is bound to a representation of the value of that variable, and different types of uncertainty are represented in different neural networks, whereas other theories suggest that multiple distinct decision-making variables are processed in a unified network (Dayan et al. 1995; Hinton and Dayan 1996; Friston 2009; Friston and Kiebel 2009).

The medial prefrontal cortex (mPFC) has been shown to be involved in the representation and reduction of a variety of uncertainty (Corbetta et al. 1991; Critchley et al. 2001; Matsumoto et al. 2003; Keri et al. 2004; Behrens et al. 2007; Rushworth and Behrens 2008; Christopoulos et al. 2009; Bach and Dolan 2012; Payzan-LeNestour et al. 2013; McGuire et al. 2014; Monosov 2017; Muller et al. 2019). In the present study, we investigated how neurons in the mPFC represent different types of uncertainty. We trained macaque monkeys in a task that had two qualitatively different types of uncertainty. The degree of uncertainty, which varied among trials, was estimated by simulating the monkey’s learning behavior or the design of the task. Neurons in the lateral prefrontal cortex (lPFC) as well as those in the mPFC were recorded for comparison. We found that activities in different groups of neurons in the mPFC and lPFC represented the two different types of uncertainty.

## Materials and Methods

### Subjects

Two male macaque monkeys (*Macaca mulatta*) weighing 7–10 kg were used (Matsumoto, Matsumoto, Abe, et al. 2007). A head holder and two recording chambers (20 mm in diameter, each), one for recordings from the mPFC in both hemispheres and the other for recordings from the left lPFC, were implanted through aseptic surgery under pentobarbital anesthesia (35 mg/kg i.p.). All procedures were approved by the RIKEN Animal Experiment Committee and were in accordance with the US NIH Guidelines for the Care and Use of Laboratory Animals.

The monkeys were seated in a primate chair inside a dark room, with their heads fixed. A cathode ray tube display was placed 57 cm from the monkey’s eyes to present a fixation point and visual stimuli. Three lever switches were placed in front of the primate chair. The gaze position was measured using an infrared system (http://staff.aist.go.jp/k.matsuda/eye/). The task was controlled, and behavioral and neuronal data were recorded by computers running a commercially available system (*Tempo for Windows*, Reflective Computing, St. Louis, MO, USA).

### Behavioral Task

The task consisted of two types of trial blocks, visual blocks and action-learning blocks, which were alternated (Matsumoto, Matsumoto, Abe, et al. 2007). In a trial of the visual block, a white fixation point (0.44° wide) was presented at the center of the display after an intertrial interval varying from 1.0 to 1.5 s. After the monkey fixated its gaze on the point and held down the central lever with the right hand for 0.5 s, a visual image (a full-colored photograph of a flower, 7° wide) was presented for 0.6 s and a drop of water was delivered at the end of the visual image presentation. The monkey had to maintain eye fixation and keep the central lever depressed until water delivery. The monkey had to release the central lever after the termination of each trial; otherwise, the next trial did not start. After three successful trials of the visual block, the task moved from a visual block to an action-learning block. As the monkeys seldom failed in the eye fixation and central lever pressing in trials of the visual blocks, the transition from a visual block to an action-learning block was practically deterministic.

**Figure 1:**
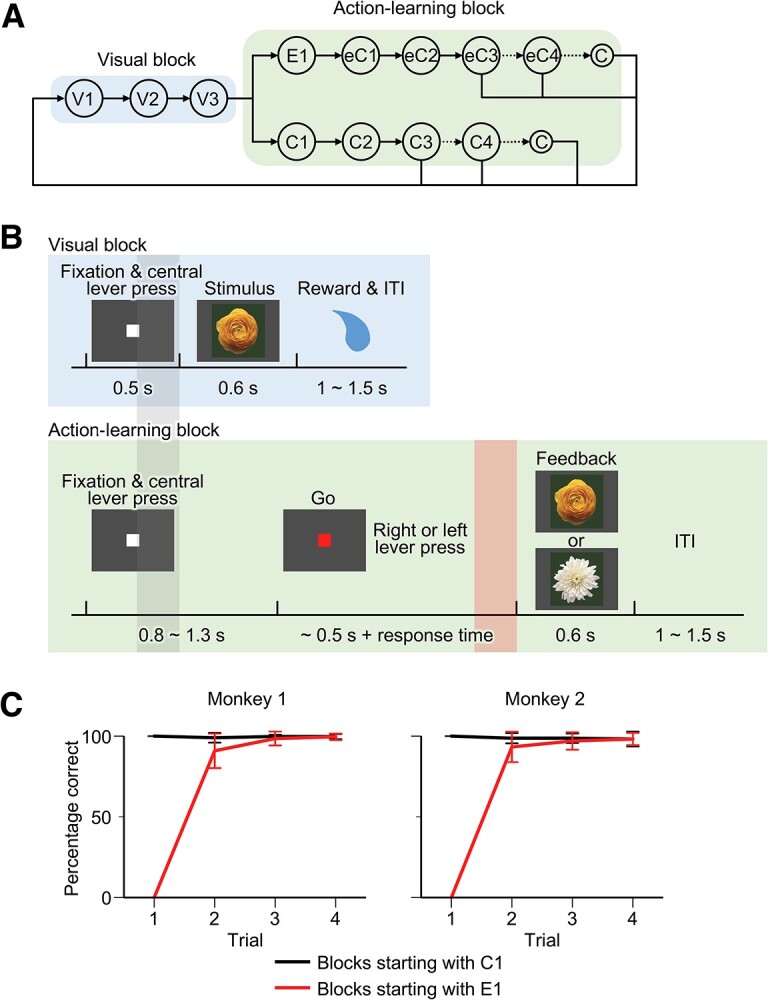
Task design and behavioral results. (*A*) Trial transition diagram. The most frequent transitions among the trial types in visual and action-learning blocks are illustrated. V1, V2, and V3 are the first, second, and third trials of visual blocks, respectively. See the main text for the abbreviations of the trial types in action-learning blocks. (*B*) Event sequences in single trials of visual and action-learning blocks. Gray and pink shading indicate the preblock-transition and prefeedback periods, respectively, in which the activities associated with the uncertainty in block transition or the uncertainty in the nature of the coming feedback were measured. (*C*) Percentage of monkeys’ correct responses in trials 1–4 of action-learning blocks. Black lines, for blocks that started with a correct trial; red lines, for blocks that started with an error trial. Error bars indicate standard deviations across blocks.

In a trial of the action-learning block, after a 1.0–1.5 s intertrial interval, the fixation point was presented at the center of the display. After the monkey fixated on it and held down the central lever from 0.8 to 1.3 s, the color of the fixation point changed to red, which instructed the monkey to initiate an action. The monkey was required to press either the left or right lever and return to the central lever within 2.0 s. At 0.5 s after the monkey returned to the central lever, a visual image was presented for 0.6 s as a feedback signal to the executed action. A correct action (see below) was followed by the visual stimulus that had been presented in the preceding visual block (positive feedback), whereas an incorrect action was followed by the image of another flower (negative feedback). The monkey continued gaze fixation and central-lever pressing until the offset of the feedback presentation. The trial was immediately aborted when either gaze fixation or central-lever pressing failed. The monkey had to release the central lever after termination of each trial to start the next trial. The correct action (left or right) was fixed within each action-learning block but pseudo-randomly changed between blocks. When the monkey repeated the correct response in three or four consecutive trials (randomly determined by the computer) in an action-learning block, the task moved to a visual block (Fig. 1*A*). When a trial was aborted by a fixation break or central-lever release during the presentation of a positive feedback signal, the trial was regarded as a correct trial, but the monkey was required to perform one more correct trial before moving to a visual block. Because of this regulation, the number of consecutive correct trials was more than four in some action-learning blocks.

Two pairs of positive and negative feedback stimuli were alternated every four repetitions of the visual and action-learning blocks. One pair was used in four repetitions of the visual and action-learning blocks, and the other pair was used for the next four repetitions. After 32 repetitions of the visual and action-learning blocks, we introduced two new pairs.

### Recordings

We recorded the action potentials of single neurons extracellularly with tungsten electrodes (impedance of 8–10 MΩ, FHC, Bowdoinham, ME), while the monkeys performed the task. The electrodes were advanced by custom-made hydraulic manipulators (Narishige, Japan) and single neuronal discharges were collected at 1 kHz using a template-matching spike discriminator (Alpha-Omega, Alpharetta, GA). The activity of the single cells was recorded from both the mPFC and the lPFC in the same sessions (Fig 7*A*,*B*) (Matsumoto, Matsumoto, Abe, et al. 2007). The recording area in the mPFC was located in the dorsal bank and fundus of the anterior part of the cingulate sulcus in both hemispheres of both monkeys (the leftmost diagram in Fig. 8). The anterior–posterior ranges of the recordings were A30-A35 in Monkey 1 and A31-A37 in Monkey 2, which were largely located anterior to the genu of the corpus callosum and the anterior tip of the arcuate sulcal inferior limb. The recording area in the lPFC was located on the lateral surface, both dorsal and ventral to the principal sulcus in the left hemispheres of both monkeys. The regions of recordings corresponded to the middle part of the anterior–posterior extent of the sulcus and ranged from A31 to A38 in Monkey 1 and A33-A38 in Monkey 2. These corresponded to the area 46 (Walker 1940; Barbas and Pandya 1989). The position and extent of recordings were determined on the basis of structural magnetic resonance images (4 T, Varian NMR Instruments, Palo Alto, CA) taken before the surgery.

### Layer Distribution of the mPFC Cells

For 13 of the 32 grid positions in the mPFC recording chamber (7/18 in Monkey 1, 6/14 in Monkey 2), the electrode tip went through all the layers of the cortex in the dorsal bank of the cingulate sulcus, which allowed us to estimate the depth position of the recording cell activities in the dorsal bank of the cingulate sulcus. In these penetrations, the electrode tip passed through the gray matter in the dorsal surface of the brain, the white matter under the dorsal surface gray matter, and the gray matter in the dorsal bank of the cingulate sulcus, finally entering the cingulate sulcus (see the leftmost diagram in Fig. 8). After being left unmoved in the white matter for ~30 min, the electrode was slowly advanced to determine the position at which action potentials were obtained. This position was regarded as the border between the white matter and gray matter in the dorsal bank of the cingulate sulcus. The border between the gray matter in the dorsal bank of the cingulate sulcus and the cingulate sulcus was determined by the last position of the action potentials, beyond which the recording continued to be silent. The border between the white matter and gray matter in the dorsal bank of the cingulate sulcus was determined every recording day, whereas the border between the gray matter in the dorsal bank of the cingulate sulcus and the cingulate sulcus was determined only a few days toward the end of recordings in the grid position. The distance between the position at which a cell was recorded and the white matter/gray matter border was divided by the average distance between the gray matter/cingulate sulcus border and the white matter/gray matter border (2169 ± 237 μm [mean ± SD] for Monkey 1 and 2061 ± 277 μm for Monkey 2) to determine the normalized depth of the cell.

### Statistical Analyses

We analyzed the cells recorded for 16–32 repetitions of visual and action-learning blocks. We selected cells that significantly increased the firing rate before the feedback presentation in trials C1 and E1 by comparing firing during the last 200 ms of the prefeedback period with firing during the initial fixation period (100–500 ms after gazing at the fixation point started) in C1 and E1 trials (*P <* 0.05, paired *t*-test). This analysis was conducted on the data of trials pooled for all four combinations of motor responses (left and right) and feedback pairs (a feedback pair A and a feedback pair B). Trials were not divided according to the executed action, because the majority (mPFC: 86% [48/56], lPFC: 86% [48/56]) of the cells with significant prefeedback activities in C1/E1 did not show the main effects of the action type (*P* < 0.05, two-way factorial ANOVA with factors of motor response and feedback pair). The feedback pair used as a factor in this analysis is the pair of positive and negative feedback stimuli, which was changed every four repetitions of the visual and action-learning blocks (see above). C1 trials were combined with E1 trials, because the activities were analyzed in the prefeedback period during which the feedback was not yet presented. Finally, trials with different feedback pairs were pooled, because the majority (mPFC: 96% [54/56], lPFC: 86% [48/56]) of the cells with significant prefeedback activities in C1/E1 did not show the main effects of the feedback pair (*P* < 0.05, the same ANOVA described above).

We determined the rising onset of the prefeedback activity in each cell by comparing the firing during the moving window of 200 ms with that during the initial fixation period (100–500 ms after the start of the gaze fixation) in C1/E1 (*P <* 0.05, paired *t*-test). The window was shifted back from the time immediately before the feedback onset in 50-ms steps and the center of the last window with which a significant difference was obtained was used as the rising onset.

The prefeedback activities decreased from C1/E1 to later trials in each action-learning block. To examine the significance of this trend in the cell population, we compared the magnitude of activities between C1/E1 and each of the later correct trial types (C2, C3, eC1, eC2, and eC3) using the Wilcoxon signed-rank test (*P* < 0.05). To avoid possible effects of random fluctuations of activity in C1/E1, we also conducted the procedure using half of the C1/E1 trials (in odd blocks in each action-outcome combination) for cell selection and the remaining half for comparison between C1/E1 and later correct trials (in even blocks in each action-outcome combination).

To estimate the uncertainty of feedback, we first determined the internal model parameters of the monkey’s selection behavior, including the prediction errors of action values and estimated action-selection probability in each trial type of action-learning blocks and the goodness of the feedback stimuli, by fitting a reinforcement learning model to the monkey’s behavior (see our previous paper Matsumoto, Matsumoto, Abe, et al. 2007 for details). We chose a “double-update” model, in which the values of both executed and nonexecuted action types are updated when the feedback is given, rather than the “single-update” model, in which only the value of the executed action is updated (Watkins and Dayan 1992), because the double-update model better fitted the behaviors of our two monkeys (see Supplementary Fig. 2 and Supplementary Note online in our previous paper Matsumoto, Matsumoto, Abe, et al. 2007). We assumed a Boltzmann selection rule for action selection, and the probability of selecting an action *a* (either left [*L*] or right [*R*]) is given by

(1) \begin{equation*} p(a)=\frac{\exp \left(\beta Q(a)\right)}{\exp \left(\beta Q(L)\right)+\exp \left(\beta Q(R)\right)}, \end{equation*}

where $Q(a)$ is the action value of action $a$. $\beta$ is the inverse temperature, which is inversely related to randomness in action selection $(\beta \ge 0)$. When the feedback was given, the values of the nonexecuted action $\overline{a}$ as well as of the executed action $a$ were updated using the following formulae:

(2) \begin{equation*} \delta Q(a)=r-Q(a) \end{equation*}

(3) \begin{equation*} Q(a)\leftarrow Q(a)+\alpha \delta Q(a) \end{equation*}

(4) \begin{equation*} Q\left(\overline{a}\right)\leftarrow Q\left(\overline{a}\right)+ i\alpha \delta Q(a), \end{equation*}

where $\delta Q(a)$ is the prediction error, $r$ is the goodness of the feedback stimulus, $\alpha$ is the learning rate $(0<\alpha <1)$ (Watkins and Dayan 1992), and $i$ is an interaction factor $(-1\le i\le 0)$. $r$ was 1 for positive feedback stimuli and ${\nu}_{\mathrm{neg}}\ (-1\le{\nu}_{\mathrm{neg}}\le 1)$ for negative feedback stimuli.

The action values were reset to 0 at the beginning of each action-learning block and sequentially changed along the series of actions within each action-learning block. We determined the parameters with which the model best fits the monkey’s actual action selections, according to a likelihood function $l(\theta |y)$ for each set of parameters ($\theta$) with particular behavioral data ($y$).

(5) \begin{equation*} l\left(\theta |y\right)=\prod p\left(a,t|\theta \right). \end{equation*}

We considered the set of parameters that provided the largest value of the likelihood function as the best-fit parameter. For $y$, we pooled, separately, for each monkey, the behavioral data of all the sessions in which we recorded neuronal activities.

For the uncertainty of the values of feedback stimuli, we calculated the standard deviation of the goodness of feedback stimuli (${SD}_{\mathrm{feedback}}$) and the expected value of the absolute prediction error of action values (${{EV}}_{\mathrm{PE}}$). ${SD}_{\mathrm{feedback}}$ in each trial type was calculated using the following formula:

(6) \begin{equation*} {SD}_{\mathrm{feedback}}=\sqrt{P_{\mathrm{correct}}{\left({r}_{\mathrm{pos}}-\overline{r}\right)}^2+{P}_{\mathrm{error}}{\left({r}_{\mathrm{neg}}-\overline{r}\right)}^2} \end{equation*}

(7) \begin{equation*} \overline{r}={P}_{\mathrm{correct}}\ast{r}_{\mathrm{pos}}+{P}_{\mathrm{error}}\ast{r}_{\mathrm{neg}}, \end{equation*}

where ${P}_{\mathrm{correct}}$ and ${P}_{\mathrm{error}}$ are the actual conditional probabilities of correct and erroneous responses in each trial type, respectively; ${r}_{\mathrm{pos}}$ is 1 (arbitrarily defined as the goodness for positive feedback stimuli), and ${r}_{\mathrm{neg}}$ is ${\nu}_{\mathrm{neg}}$, which was determined for each monkey as a behavioral parameter as described above (see the values in Supplementary Table 2).

Next, we examined the correlation between changes in the prefeedback activities and those in the uncertainty of the values of feedback stimuli: (i) the activity of each cell in each trial type was normalized by the cell’s maximal activity (among the trial types) after the cell’s minimal activity (among the trial types) was subtracted from the activities in each trial type; (ii) for each monkey, the value of ${SD}_{\mathrm{feedback}}$ in each trial type was normalized by the maximal value of ${SD}_{\mathrm{feedback}}$ (among the trial types) after the minimal value of ${SD}_{\mathrm{feedback}}$ was subtracted from values in each trial type; and (iii) the distribution of the normalized activities of cells was compared for each trial type, with the normalized values of ${SD}_{\mathrm{feedback}}$, for each monkey, using Wilcoxon signed-rank text. We also performed Bayesian hypothesis tests using the multiplatform open-source program JASP (Jeffreys’s Amazing Statistics Program; https://jasp-stats.org): the Bayes factor *BF_10_* was calculated using the Bayesian Wilcoxon signed-rank test and default effect size priors (Cauchy scale 2^0.5^/2) (Keysers et al. 2020; van Doorn et al. 2020).

We also calculated the expected values of absolute prediction errors (${{EV}}_{\mathrm{PE}}$) as a biologically plausible approximation of the uncertainty of the values of feedback stimuli, which would be provided by the feedback in the trial. ${{EV}}_{\mathrm{PE}}$ in each trial type was calculated using the following formula:

(8) \begin{equation*} {{EV}}_{\mathrm{PE}}={P}_{\mathrm{correct}}\left|\delta{Q}_{\mathrm{correct}}\right|+{P}_{\mathrm{error}}\left|\delta{Q}_{\mathrm{error}}\right|, \end{equation*}

where ${P}_{\mathrm{correct}}$ and ${P}_{\mathrm{error}}$ are the estimated conditional probabilities of correct and erroneous responses in each trial type, respectively, and $\delta{Q}_{\mathrm{correct}}$ and $\delta{Q}_{\mathrm{error}}$ are the expected prediction errors for correct and erroneous responses in each trial type, respectively (see the values in Supplementary Table 2). We examined the correlation between changes in the prefeedback activities and those in ${{EV}}_{\mathrm{PE}}$ in the same way as described above for the correlation between changes in the prefeedback activities and those in ${SD}_{\mathrm{feedback}}$.The task probabilistically moved from an action-learning block to a visual block after C3, eC3, C4, or eC4. In the trials after C3, eC3, C4, and eC4 (with the four groups of trials pooled), we compared the firing rate during the 200-ms period starting at 300 ms after the eye fixation and central lever pressing started with the firing rate during the 400-ms period immediately before the fixation and central lever pressing started (*P <* 0.05, paired *t*-test). The end of the former window was the time at which the monkey could find whether or not the block transition occurred: a visual stimulus replaced the fixation point when a visual block started, whereas the fixation point remained when the action-learning block continued. Therefore, we named the window the “pretransition period.” For the population of cells that showed a significantly higher firing rate in the pretransition period than in the firing late before the start of the eye fixation and central lever pressing in the trials after Ce, eC3, C4, and eC4, we compared the firing rate during the pretransition period in the trials after C3, eC3, C4, and eC4 (pooled) with the firing rate in the corresponding window in the earlier trials after C1, eC1, C2, or eC2 (for each of the four types separately), and with the firing rate in the corresponding window in the later trials in the visual block (V2 and V3, respectively), by Wilcoxon signed-rank test (*P* < 0.05). To avoid possible effects of random fluctuations of activity in the trials after C3, eC3, C4, and eC4, we also conducted the procedure using half of the trials after C3, eC3, C4, or eC4 (odd trials) for cell selection and the remaining half for the comparison with the other trials.

We analyzed the direct effects of the presentation of visual feedback stimuli in C1/E1 trials on neuronal activity by comparing the firing rate after the onset of feedback presentation (100–400 ms after the feedback onset) with that during the 400 ms immediately before the feedback onset by paired *t*-test (*P <* 0.05). This comparison was conducted separately for the positive and negative feedback. For the cells that increased the firing rate after the onset of either positive or negative feedback, we further examined whether the firing rate after the feedback onset was different between positive and negative feedback by *t*-test (*P <* 0.05).

We analyzed the direct effects of the presentation of visual stimuli in V1 trials on neuronal activity by comparing the firing rate after the onset of visual stimulus presentation (100–400 ms after the stimulus onset) with that during the 400 ms immediately before the stimulus onset by paired *t*-test (*P <* 0.05) in V1. For the cells that increased the firing rate after the onset of the visual stimulus, we further examined whether the firing rate after the visual stimulus onset was different between V1 and V3. We conducted a two-way factorial ANOVA (*P* < 0.05) with factors of trial type (V1/V3) and stimulus (one or the other).

## Results

Two macaque monkeys performed a task in which visual and action-learning blocks were alternated (Fig. 1*A*,*B*) (Matsumoto, Matsumoto, Abe, et al. 2007). In the visual block, the presentation of a visual image (a full-colored photograph of a flower) was followed by a water reward. After three trials of the visual block, the task was moved to the action-learning block, in which the monkey had to make either a left-lever press or right-lever press. As the correct side of the response was randomly determined by the computer in each action-learning block, the monkey had to learn it by trial and error. The feedback in the action-learning block was a visual stimulus. The stimulus presented in the preceding visual block indicated that the action was correct, while the image of another flower indicated that the action was wrong. When the monkey had repeated the correct response in three or more consecutive trials (see Materials and methods) in an action-learning block, the task probabilistically moved to a visual block (Fig. 1*A*).

Thus, there were two types of uncertainty in the task. One type of uncertainty existed in the content of visual feedback given in early trials of the action-learning blocks, whereas the other type of uncertainty existed in the timing of transition from action-learning to visual blocks.

### Behavioral Results

The monkeys quickly learned the correct action in each action-learning block (Fig. 1*C*) (Matsumoto, Matsumoto, Abe, et al. 2007). The percentage of correct actions in the first trial of action-learning blocks was at chance level (56 ± 8% [mean ± SD] for Monkey 1 and 50 ± 3% for Monkey 2). The average percentage correct was over 90% in the second trial and remained at this high level in the subsequent trials, while the percentage correct in the trial after an incorrect initial trial was lower than that in the trial after a correct initial trial. Therefore, the sequence of trials was, in most blocks, either a correct initial trial (C1) followed by consecutive correct trials (C2, C3, …) or an incorrect initial trial (E1) followed by consecutive correct trials (eC1, eC2, eC3, ….) (Fig. 1*A*). We concentrated on the trials of these two sequences for the analyses of neuronal activities in action-learning blocks because the number of trials of other trial types was too few per cell. Other trials were also included when we analyzed the selection behavior of the monkeys (Supplementary Table 1).

### Representation of Uncertainty Related to the Coming Feedback

**Figure 2:**
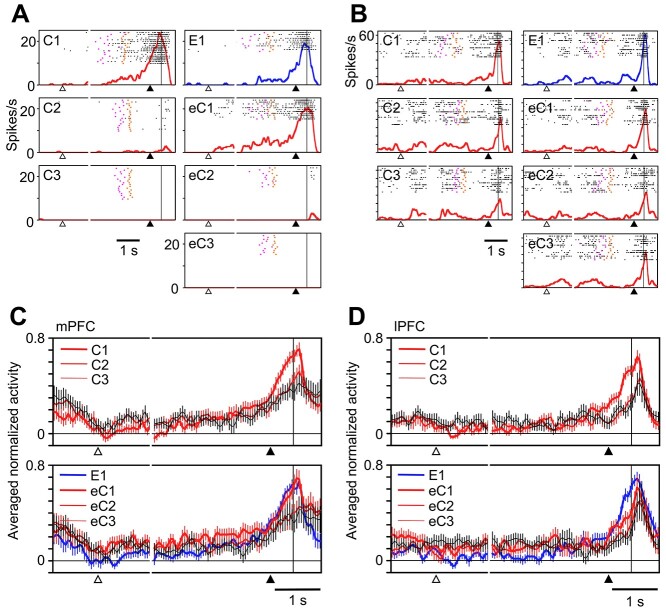
Prefeedback activities in the mPFC and lPFC. (*A*, *B*) Activities in an example mPFC cell (*A*) and lPFC cell (*B*). (*C*, *D*) Activities in the mPFC cell population (*C*) and lPFC cell population (*D*). The bin width is 50 ms. The activity graphs are aligned with the onset of the fixation period (in the left of the short gaps) or with the onset of the visual feedback stimulus (in the right of the short gaps). The open triangles below the abscissa indicate the time when the monkey depressed the central lever and started to gaze at the fixation point. The filled triangles indicate the time when the monkey returned to the central lever. The vertical lines right to the filled triangles indicate the onset of the feedback. The activities in the trials with positive feedback stimuli are shown in red and those in the trials with negative feedback are shown in blue. For the population activities, the activity in each bin was subtracted by the averaged activity in the 400-ms window starting 100 ms after the start of gaze and central lever pressing in the C1/E1 trials, normalized by the peak activity in individual cells, and then averaged across cells. The error bars indicate the standard error of mean.

In order to find neural activities associated with the uncertainty of the coming feedback, we examined PFC cell activities during the last 200 ms before the feedback presentation (the prefeedback period) in the first (C1/E1) trials of action-learning blocks. Because the feedback was presented with a fixed delay from the end of the action execution, the monkeys likely anticipated the time of feedback presentation. Of the 351 cells recorded from the mPFC and 396 cells recorded from the lPFC, 56 mPFC cells (16%) and 56 lPFC cells (14%) showed significantly higher firing rates during the prefeedback period than during the initial fixation period (400 ms immediately following the fixation start) in C1/E1 trials (*P <* 0.05, paired *t*-test). We refer to these activities as “prefeedback activities.” As shown for two example cells recorded from the mPFC (Fig. 2*A*) and lPFC (Fig. 2*B*), the prefeedback activities gradually increased their firing rate toward the onset of the feedback stimulus (vertical line).

The activities in the prefeedback period significantly decreased from C1/E1 to subsequent trials, in which the probability of positive feedback was higher. This was true in both the mPFC population (*P* < 0.001 in each of C1/E1 vs. C2, C3, eC2, and eC3, but *P =* 0.11 in C1/E1 vs. eC1, Wilcoxon signed-rank test) (Fig. 2*C*) and the lPFC population (*P* < 0.001 in C1/E1 vs. C2, C3, eC2, and eC3 and *P* = 0.002 in C1/E1 vs. eC1) (Fig. 2*D*). To confirm that these differences were not merely caused by random fluctuation of activities in the C1/E1 trials through its influence on the selection of cells with significant prefeedback activities in C1/E1, we used half of the C1/E1 trials to determine the significance of responses and the remaining half for the comparison between C1/E1 and later correct trials. Even in this analysis, the activities in C1/E1 were larger than those in the later correct trials in mPFC (44 cells, *P* = 0.001 in C1/E1 vs. C2, *P* = 0.002 in C1/E1 vs. C3, and *P* < 0.001 in C1/E1 vs. eC2 and eC3, Wilcoxon signed-rank test), except eC1 (*P* = 0.37 in C1/E1 vs. eC1, Wilcoxon signed-rank test) and in lPFC (28 cells, *P* < 0.001 in C1/E1 vs. C2, C3, and eC2, *P* = 0.004 in C1/E1 vs. eC1, and *P* = 0.01 in C1/E1 vs. eC3).

### Earlier Rising Onset in the mPFC

**Figure 3:**
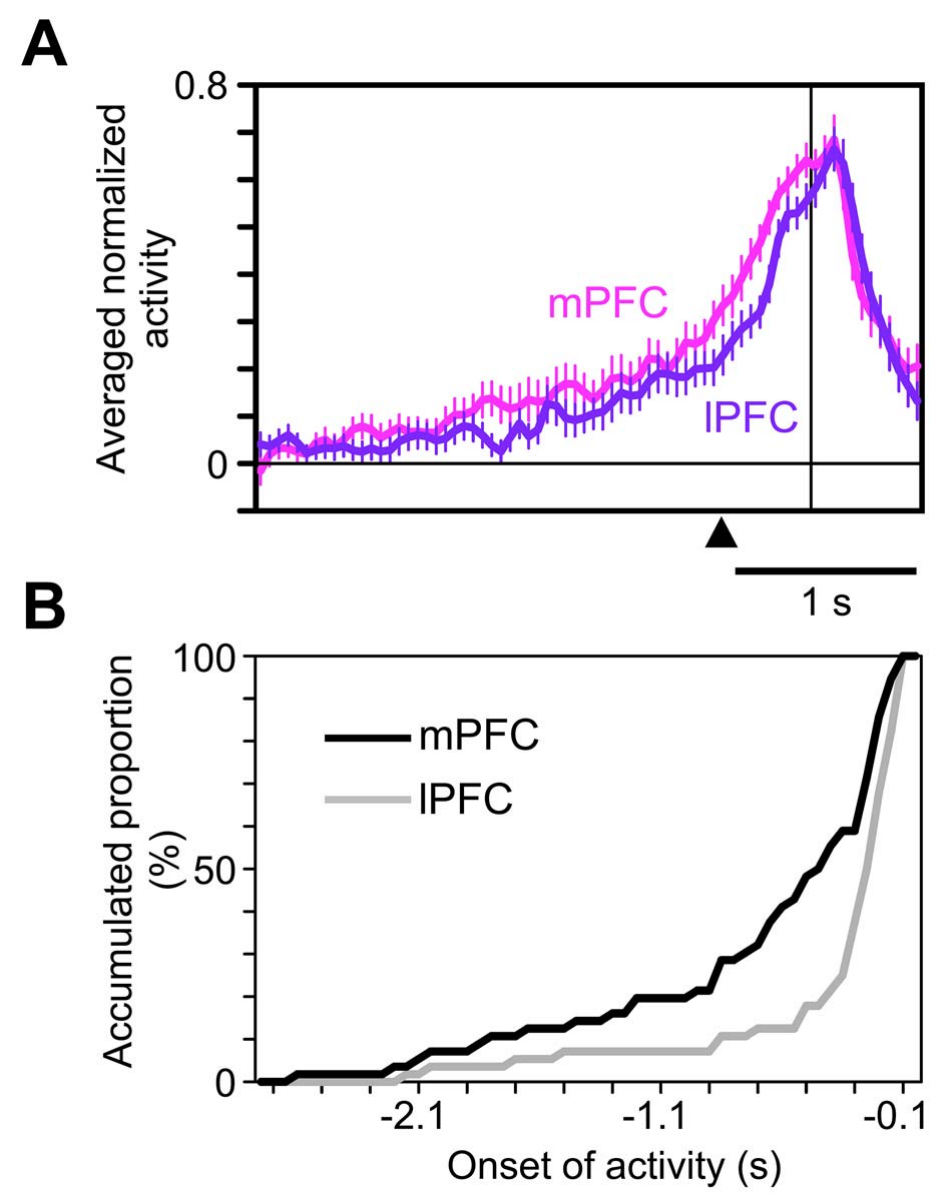
The prefeedback activities in the C1/E1 trials. (*A*) Averaged normalized prefeedback activities of the mPFC and lPFC cell populations in the 1st trial (C1 or E1) of the action-learning block. The activities were aligned at the onset of feedback (vertical line). The filled triangle indicates the time when the monkey returned to the central lever. (*B*) Cumulative distributions of the rising onset of the prefeedback activities in C1/E1 in the mPFC (black line) and lPFC (gray line).

The prefeedback activities in the first (C1/E1) trials started earlier in the mPFC than in the lPFC in individual trials (Fig. 3*A*). We determined the rising onset (the time when the activity reached a significantly higher level than that in the initial fixation period) in C1/E1 in individual cells with significant prefeedback activities in C1/E1. The mean rising onset was 663 ms before the onset of the feedback stimulus in the mPFC, whereas the mean rising onset was 372 ms before the onset of the feedback stimulus in the lPFC. The difference between the means in the mPFC and lPFC was statistically significant (*P* < 0.001, Mann–Whitney U-test) (Fig. 3*B*). The difference in the estimated rising onset was not due to a higher baseline activity of the lPFC cells compared with the mPFC cells; the discharge rate during the initial fixation period was not significantly different between the two areas (*P* = 0.23, Mann–Whitney U-test), and the baseline activity was numerically lower in the lPFC cells than in the mPFC cells.

### Correlation of the Changes in the Prefeedback Activity with Those in the Estimated Strength of Uncertainty over the Course of Action Learning

**Figure 4:**
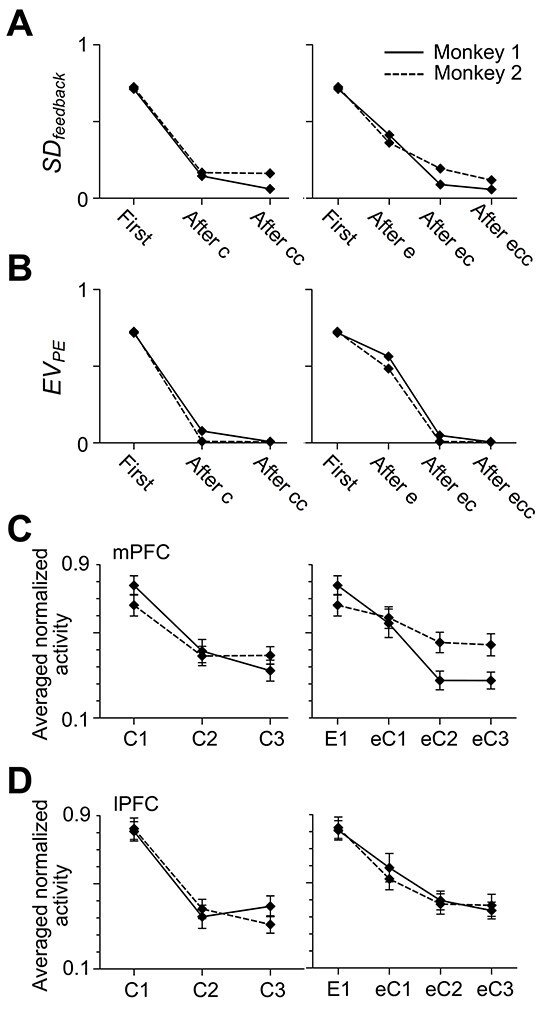
Changes of the uncertainty of the coming feedback and the prefeedback activities along the course of action learning. (*A*, *B*) Changes of the standard deviation of feedback values (*A*), the expected values of absolute prediction errors (*B*). Note that the trial types are labeled as “first,” “after c,” “after cc,” “first,” “after e,” “after ec,” and “after ecc,” instead of C1, C2, C3, E1, eC1, eC2, and eC3, because the labeling is based on the past trials that had already occurred up to the point in time when the feedback was about to be given. (*C*, *D*) Changes of the prefeedback activities in the mPFC (*C*) and lPFC (*D*) cells with significant prefeedback activities in C1/E1. The prefeedback activities were normalized by the maximum activities in each cell and averaged across all the cells with significant prefeedback activities in C1/E1 recorded from each monkey. The error bars indicate the standard error of mean across cells.

The decrease in the prefeedback activities from the first (C1/E1) to later trials might be correlated with the decrease in uncertainty of the feedback. To quantitatively examine the relationship between them, we calculated, from the monkey’s selection behavior, two values as the uncertainty: the standard deviation of the values of feedback stimuli (${SD}_{\mathrm{feedback}}$) (Fig. 4*A*) and the expected value of the absolute prediction error of action value (${{EV}}_{\mathrm{PE}}$) (Fig. 4*B*, see Materials and methods). The standard deviation is a mathematically normative value used to represent uncertainty, but its calculation includes the square and square root operations, which are biologically implausible. The expected value of the absolute prediction error, which can be calculated by summing the absolute value of prediction errors with weights of probability, approximates the standard deviation (Soltani and Izquierdo 2019).

The changes of the prefeedback activities in the mPFC and lPFC matched those of ${SD}_{\mathrm{feedback}}$ (Fig. 4*C*,*D*, see Materials and methods): the normalized prefeedback activities (of the cells with significant prefeedback activities in C1/E1) did not significantly differ from the normalized ${SD}_{\mathrm{feedback}}$ in the mPFC or in the lPFC (Wilcoxon signed-rank test, Table 1). Furthermore, the Bayes factor *BF_10_* (Keysers et al. 2020; van Doorn et al. 2020) took values between 1/3 and 1/10 for most trial types and never exceeded 3 in both the mPFC and the lPFC (Bayesian Wilcoxon signed-rank test, Table 2), which moderately supports the hypothesis that the normalized prefeedback activities matched the normalized ${SD}_{\mathrm{feedback}}$. The changes of the prefeedback activities in the mPFC and lPFC matched those of ${{EV}}_{\mathrm{PE}}$ as well (Fig. 4*C*,*D*): the normalized prefeedback activities did not significantly differ from the normalized ${{EV}}_{\mathrm{PE}}$ in the mPFC or in the lPFC (Wilcoxon signed-rank test, Table 1). The Bayes factor *BF_10_* took values between 1/3 and 1/10 for most trial types and never exceeded 3 in both the mPFC and the lPFC (Bayesian Wilcoxon signed-rank test, Table 2).

**Table 1:** Significance of the difference between the normalized prefeedback activities and the estimated strength of uncertainty, over the course of action learning: Wilcoxon signed-rank test. *P*-values are shown for each trial type

			C1/E1	C2	C3	eC1	eC2	eC3
SD_feedback_	mPFC	Monkey 1	0.62	0.23	0.70	0.95	0.36	0.61
		Monkey 2	0.69	0.46	0.73	0.09	0.74	0.52
	IPFC	Monkey 1	0.97	0.31	0.30	0.61	0.50	0.59
		Monkey 2	0.27	0.90	0.17	0.52	0.32	0.09
EV_PE_	mPFC	Monkey 1	0.62	0.14	0.70	0.24	0.33	0.61
		Monkey 2	0.69	0.63	0.94	0.61	0.35	0.52
	IPFC	Monkey 1	0.97	0.42	0.30	0.18	0.53	0.59
		Monkey 2	0.27	0.34	0.46	0.06	0.12	0.09

**Table 2:** Significance of the difference between the normalized prefeedback activities and the estimated strength of uncertainty, over the course of action learning: Bayesian Wilcoxon signed-rank test. Bayes factor *BF_10_* is shown for each trial type

			C1/E1	C2	C3	eC1	eC2	eC3
SD_feedback_	mPFC	Monkey 1	0.23	0.71	0.28	0.23	0.25	0.22
		Monkey 2	0.19	0.21	0.19	1.24	0.25	0.32
	IPFC	Monkey 1	0.26	0.31	0.47	0.23	0.38	0.25
		Monkey 2	0.20	0.20	0.24	0.30	0.42	1.55
EV_PE_	mPFC	Monkey 1	0.22	1.55	0.29	0.65	0.25	0.22
		Monkey 2	0.19	0.21	0.20	0.20	0.42	0.31
	IPFC	Monkey 1	0.24	0.25	0.46	0.76	0.36	0.23
		Monkey 2	0.21	0.44	0.20	2.34	1.05	1.61

### Representation of Uncertainty in the Transition from an Action-Learning Block to a Visual Block

**Figure 5:**
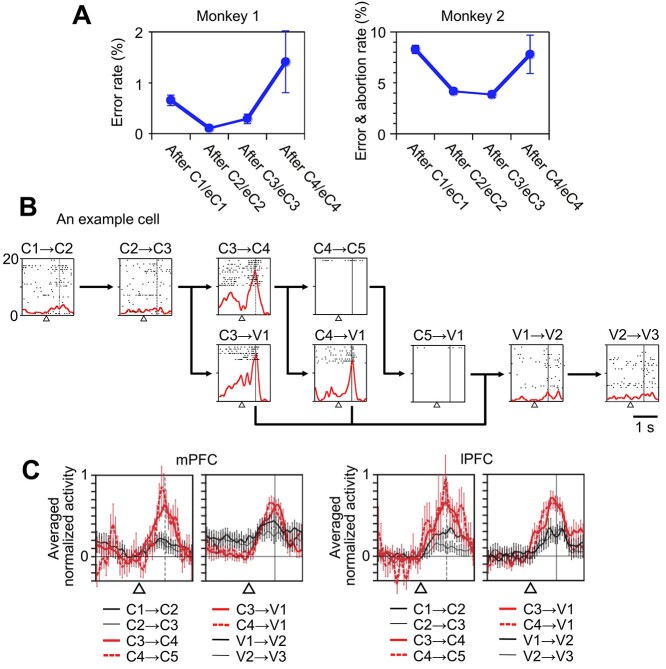
Preblock-transition activities. (*A*) Performance deterioration in the later parts of the action-learning blocks. After C4 and eC4, the ratio of the erroneous responses in which the incorrect lever was chosen in the trials increased in Monkey 1 (left), and the ratio of trials in which either the incorrect lever was chosen or the trial was aborted by a fixation break or by an early central-lever release increased in Monkey 2 (right). The ratios were averaged over all recording sessions. The error bars indicate the standard error of mean across sessions. (*B*) Activities in an example mPFC cell that showed gradually increasing activity during the fixation period after C3, eC3, C4, and eC4, where a probabilistic transition from an action-learning block to a visual block occurred. C1 → C2 represents the transition from C1 to C2 or eC1 to eC2; C3 → V1 represents the transition from C3 or eC3 to V1, and so on. (*C*) Population activities of the cells with significant preblock-transition activities in the mPFC and lPFC during the fixation period. The activities that occurred when the block transition might occur are indicated in red. The activity in each bin was subtracted by the averaged activity in the 400-ms window immediately before the gaze fixation and central lever pressing started after C3, eC3, C4, and eC4 trials. The activity in each bin was normalized by the peak activity in each cell and then averaged across cells. The error bars indicate the standard error of mean across cells. In (*B*) and (*C*), the open triangles indicate the time when the monkey depressed the central lever and started to gaze at the fixation point, and the vertical solid (in *B*) and dashed (in *C*) lines indicate the expected or actual onset of the visual stimulus, respectively. The activity graphs are aligned with the onset of the fixation period.

To determine the neural activities associated with the uncertainty in block transition, we examined the activities of the PFC cells at the block transition from an action-learning block to a visual block. The transition from an action-learning block to a visual block was probabilistic in that it occurred after three, four, or more consecutive correct trials (see Materials and methods), whereas the transition from a visual block to an action-learning block always occurred after three trials of the visual block. The probabilities of block transition from an action-learning block to a visual block and the entropies calculated from them are shown in Supplementary Table 1. After four trials, task performance deteriorated in both monkeys (Fig. 5*A*). The proportion of erroneous trials (Monkey 1) or the summed proportion of erroneous trials and trials aborted by fixation break or early central-lever release (Monkey 2) was significantly larger in the trials after C4 and eC4 than in those after C2 and eC2 (*P* = 0.017 in Monkey 1, *P* = 0.033 in Monkey 2, one-tailed paired *t*-test). These results suggest that the monkeys anticipated the transition, and their performance deteriorated when the anticipated transition did not occur.

The uncertainty about block transition was resolved at 0.5 s after the start of the eye fixation and central lever pressing. When the transition occurred, the fixation point was replaced with a visual stimulus. When the transition did not occur, the fixation point remained. Therefore, we compared the firing rate during the 200-ms period immediately before this time (pretransition period) with that during the 400-ms period immediately before the eye fixation started. The firing rate in the pretransition period was significantly higher than that before the fixation onset in 15% of the mPFC cells (52/351) and 12% of the lPFC cells (48/396) in the trials after C3, C4, eC3, and eC4 (*P <* 0.05, paired *t*-test). We refer to these activities as “preblock-transition activities” (Fig. 5*B*,*C*).

The activities of these cells in the corresponding window were significantly smaller in the earlier trials of action-learning blocks, in which the monkeys were unlikely to anticipate the block transition (for the mPFC, *P* < 0.001 for C1, C2, C3, E1, eC1, eC2, and eC3; for the lPFC, *P* < 0.001 for C1, C2, C3, E1, eC1, eC2, and eC3, Wilcoxon signed-rank test). The activities of the cells in the corresponding window were also significantly smaller in later trials of visual blocks (V2 and V3), in which the monkey fully anticipated the appearance of the visual stimulus (for the mPFC, *P* < 0.001 for V2 and V3; for the lPFC, *P* < 0.001 for V2 and V3, Wilcoxon signed-rank test). These results were confirmed even after correcting for the possible link between cell selection and testing. We reselected cells with significant preblock-transition activities based on half of the trials and compared the activity using the remaining half of the trials. In this analysis, the activities during the pretransition period after C3, C4, eC3, or eC4 were significantly larger than those in the other trials, both in the mPFC (44 cells, *P* < 0.001 for C1, C2, C3, E1, eC1, eC2, eC3, and V3; and *P* = 0.006 for V2, Wilcoxon signed-rank test) and the lPFC (32 cells, *P* < 0.001 for C1, C2, C3, E1, eC1, eC2, and eC3, *P* = 0.006 for V2 and V3).

### Representation of Different Types of Uncertainty by Distinct Neural Populations

**Figure 6:**
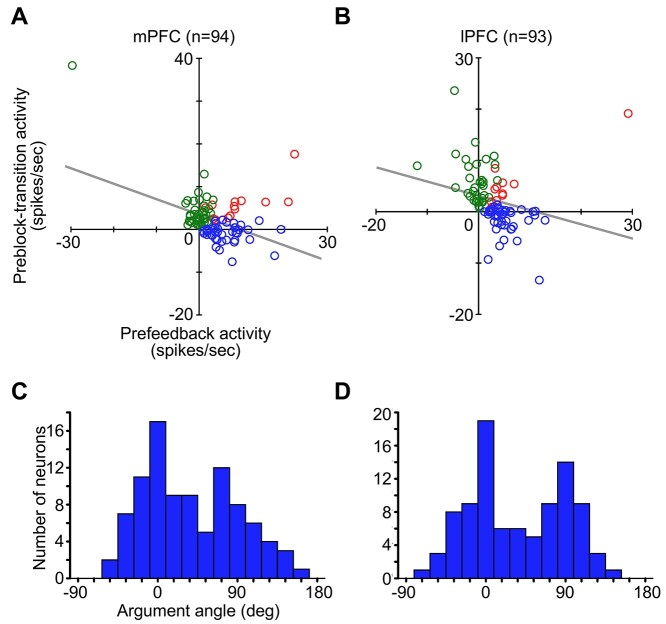
Comparison between the magnitudes of prefeedback activities in C1/E1 and preblock-transition activities. (*A*) Scatterplot of preblock-transition activities (ordinate) against prefeedback activities (abscissa) in mPFC cells. The cells with significant prefeedback activities alone, those with significant preblock-transition activities alone, and those with both significant prefeedback and preblock-transition activities are plotted by blue, green, and red circles, respectively. The prefeedback activities were subtracted by the initial fixation-period activity in the 400-ms window (100–500 ms after gazing at the fixation point started) in C1/E1 trials. The preblock-transition activities were subtracted by the averaged baseline activity in the 400-ms window immediately before the gaze fixation and the central lever pressing started after C3, eC3, C4, and eC4 trials. (*B*) Scatterplot of the preblock-transition activities against prefeedback activities in lPFC cells. (*C*, *D*) The distribution of the argument angle of each cell’s vector. The bin width of 18° was determined using the Freedman–Diaconis rule for the mPFC histogram (*C*) and was also applied to the lPFC histogram (*D*) to match them.

The prefeedback activities in the first (C1/E1) trials of action-learning blocks and preblock-transition activities occurred in largely different cell groups. Most of the cells with significant prefeedback activities in C1/E1 (42/56 in the mPFC and 45/56 in the lPFC) did not show significant preblock-transition activities (blue plots in Fig. 6*A*,*B*, Supplementary Fig. 1*A*). Conversely, most of the cells with significant preblock-transition activities (38/52 in the mPFC and 37/48 in the lPFC) did not show significant prefeedback activities in C1/E1 (green plots in Fig. 6*A*,*B*, Supplementary Fig. 1*B*). Only 14 mPFC cells and 11 lPFC cells showed both prefeedback and preblock-transition activities (red plots in Fig. 6*A*,*B*). For the cells with significant prefeedback activities in C1/E1 or significant preblock-transition activities, the prefeedback activities in C1/E1 negatively correlated with preblock-transition activities in both the mPFC (Fig. 6*A*, Spearman’s *r* = −0.34 *P* = 0.0007) and the lPFC (Fig. 6*B*, *r* = −0.59 *P* = 7.3 × 10^−10^), indicating that the cells with larger prefeedback activities tended to show smaller preblock-transition activities and the cells with larger preblock-transition activities tended to show smaller prefeedback activities. To examine further how separately the two types of uncertainty are represented by different neuronal populations, we calculated the argument angle of the vector given by the prefeedback and preblock-transition activity pairs for each of the cells that showed significant prefeedback activities or preblock-transition activities, in the space of the two activities. The distributions of the argument angles are shown in the histograms (Fig. 6*C*,*D*) with the width of the bins determined using the Freedman–Diaconis rule (Freedman and Diaconis 1981). They appeared to have two peaks: one around 0° corresponding to the positive range in the abscissa, and the other around 90° corresponding to the positive range in the ordinate, in the mPFC (Fig. 6*C*) and lPFC (Fig. 6*D*).

### Relationship of Prefeedback Activities with Transient Responses after Feedback and Relationship of Preblock-Transition Activities with Activities after Block Transition

We previously reported prominent neuronal responses in the mPFC and lPFC after feedback (Matsumoto, Matsumoto, Abe, et al. 2007). These responses were different from the prefeedback activities: the former transiently occurred after the feedback, whereas the prefeedback activities gradually grew toward the onset of feedback. Most of the cells with significant prefeedback activities in C1/E1 (40/56 in the mPFC and 37/56 in the lPFC) did not show further increases in firing after the feedback onset for either positive or negative feedback (*P >* 0.05, paired *t*-test, see Materials and methods), as illustrated for the two example cells in Fig. 2*A*,*B*. The remaining 16 (29%) mPFC and 19 (34%) lPFC cells showed a further increase in firing after positive or negative feedback onset (*P <* 0.05, paired *t*-test). The numbers of prefeedback cells that showed the feedback responses representing positive, negative, and absolute prediction errors were 4, 4, and 8 in the mPFC and 1, 7, and 11 in the lPFC, respectively.

In our previous paper (Matsumoto, Matsumoto, Tanaka 2007), we reported that some cells in the lPFC and mPFC specifically responded to the appearance of visual stimuli in the first trials of visual blocks: their responses to the visual stimuli in the first trials of visual blocks were significantly larger than those in the subsequent trials of visual blocks. These activities occurred after the stimulus onset, while the preblock-transition activities occurred before the timing of possible stimulus onset, although the preference for the first trial compared with the following trials in the visual blocks was common. The overlap between the cells with the two types of activities was small; in only a minority of the cells with significant preblock-transition activities (9/52 in mPFC and 6/48 in lPFC), the firing rate further increased after stimulus onset (*P <* 0.05, paired *t*-test) and this poststimulus response decreased from V1 to V3 (*P <* 0.05, paired *t*-test).

### Distribution of Cells with Prefeedback Activities in C1/E1 and those with Preblock-Transition Activities

**Figure 7:**
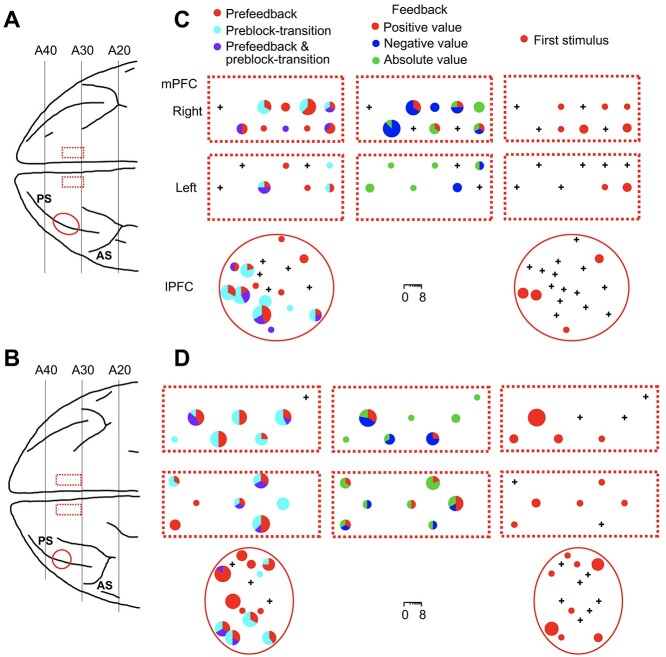
Distribution of cells with different types of activities. (*A*, *B*) The positions of the mPFC (dotted lines) and lPFC (solid ellipse) recordings in Monkey 1 (*A*) and Monkey 2 (*B*) shown on the top view of the brain. PS, principal sulcus; AS, arcuate sulcus. (*C*, *D*) Pie charts indicating the numbers and proportions, in individual electrode penetration positions, of the cells with significant prefeedback activities in C1/E1 (prefeedback), cells with significant preblock-transition activities (preblock-transition), cells responding to the feedback in action-learning blocks (feedback), and cells responding to the first appearance of visual stimulus in visual blocks (first stimulus). The cells responding to the feedback are divided into those representing the positive value of feedback (positive), those representing the negative value of feedback (negative), and those representing the absolute value of feedback (absolute value). + indicates the penetrations in which none of these activities was observed in recorded cells.

The cells with prefeedback activities in C1/E1 were locally intermingled with the cells with preblock-transition activities and the cells responding to the first appearance of visual stimulus in visual blocks in individual tracks of electrodes in both the mPFC and lPFC (Fig. 7*C*,*D*, left and right). They were also intermingled with the cells responding to feedback in action-learning blocks and in individual tracks of electrodes in the mPFC (Fig. 7*C*,*D*, middle column).

**Figure 8:**
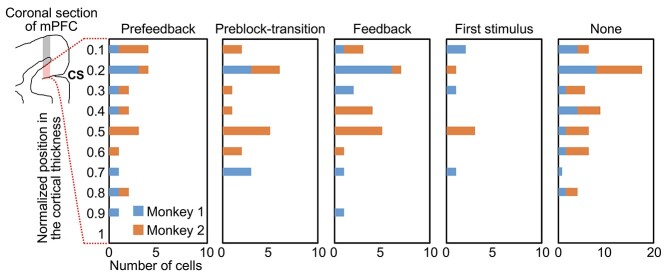
Layer distributions of cells with different types of activities in the mPFC. The *y*-axis indicates the distance of the recording position from the white matter-gray matter (W/G) boundary normalized by the total thickness of the gray matter, both along the penetration in red shaded area of mPFC section shown at leftmost; 0 indicates the W/G boundary of the deep layer (upper dashed line from the mPFC section) and 1 indicates the border of the superficial layer closest to the cingulate sulcus (lower dashed line from the mPFC section). The rightmost histogram indicates the number of cells that showed none of the activities. CS, cingulate sulcus.

For the penetrations that went through the whole cortical thickness of the gray matter in the dorsal bank of the cingulate sulcus, the positions of the recorded mPFC cells along the cortical thickness were estimated (see Materials and methods). Cells with prefeedback activities in C1/E1, cells with preblock-transition activities, cells responding to the feedback, and cells responding to the first appearance of visual stimulus in visual blocks were distributed over the cortical layers (*P* = 0.70, Fisher’s exact test) with no clear bias (Fig. 8).

## Discussion

In the present study, we found two distinctive types of cell activities representing the two different types of uncertainty in the mPFC and lPFC. One type of activity appeared before the onset of visual feedback in the early trials of action-learning blocks, in which the monkey was uncertain about the correctness of an executed action and then the type of feedback (positive/negative) (prefeedback activities in C1/E1). The magnitude of these activities correlated with the estimates of the uncertainty of the feedback value. The second type of activity appeared before the timing of the first indication of the transition from an action-learning block to a visual block (preblock-transition activities). The appearance of a visual stimulus during the initial eye fixation period indicated the transition, whereas the absence of a visual stimulus indicated the continuation of the action-learning block. The magnitude of these activities correlated with the uncertainty of the visual stimulus appearance. The two types of activities were observed in two largely nonoverlapping groups of cells in both the mPFC and the lPFC. Thus, we suggest that the mPFC and lPFC discriminate the type of uncertainty by representing different types of uncertainty with activities in different groups of cells.

To measure the uncertainty of the feedback, we calculated the standard deviation of feedback (${{SD}}_{\mathrm{feedback}}$) and the expected value of the absolute prediction error of action value (${{EV}}_{\mathrm{PE}}$). The latter, which can be calculated with biologically plausible variables (prediction errors) and operations (weighted sum), approximates the former (Soltani and Izquierdo 2019). The representation of the expected value of the absolute prediction error in the PFC cells should have been developed by repeatedly experiencing the prediction errors in various steps along action-learning. Several groups of researchers, including ours, have previously found mPFC cells whose responses to the feedback closely matched the magnitude of prediction errors of action values given by the feedback for the executed action (Ito et al. 2003; Seo and Lee 2007; Matsumoto, Matsumoto, Abe, et al. 2007; Quilodran et al. 2008). Some of the cells represented positive prediction errors, others represented negative prediction errors, and the remaining represented the absolute value of prediction errors (Ito et al. 2003; Seo and Lee 2007; Matsumoto, Matsumoto, Abe, et al. 2007; Quilodran et al. 2008). The cells with prefeedback activities in C1/E1 were intermingled with these feedback-responsive cells in the mPFC. This mixed distribution might help the former cells learn to represent the expected amount of prediction error by locally gathering the signals from the latter cells. Some theories assume that uncertainty about the value of a particular variable is bound to the representation of the value of that variable (Dayan et al. 1995; Hinton and Dayan 1996; Friston 2009; Friston and Kiebel 2009). This mixed distribution is consistent with these theories. We also found feedback-responsive cells in the lPFC, but their activities correlated with stimulus novelty rather than prediction errors (Matsumoto, Matsumoto, Abe, et al. 2007). The information about the uncertainty of the feedback in the lPFC cells might originate from the mPFC, because their activities rose later than the mPFC cells (Fig. 3).

The appearance of a visual stimulus during the initial eye fixation period was the first indication of a transition from a visual block to an action-learning block. The preblock-transition activities in the mPFC and lPFC appeared before the timing of probabilistic appearance of a visual stimulus in the trials after C3/eC3 and C4/eC4 trials but did not appear in the corresponding period in the trials after C1/eC1 and C2/eC2, in which a visual stimulus never appeared, or in the later trials of visual blocks (V2 and V3), in which a visual stimulus always appeared. In the trials after the C3/eC3 and C4/eC4 trials, the monkey had to simultaneously prepare both task sets: one to execute the correct action when no visual stimulus had appeared, and the other to remember the visual stimulus as the positive feedback in the following action-learning block when a visual stimulus had appeared. We did not find cells showing activities at the opposite transition from a visual block to an action-learning block, which regularly occurred after three trials in the visual block. This asymmetry suggests that the preblock-transition activities were associated with the uncertainty in block transition rather than the mere expectation of the coming block.

The anticipatory activity for the block transition should have been developed by repeatedly experiencing a probabilistic transition from an action-learning block to a visual block. We have previously found cells that specifically responded to the first visual stimulus appearance in the visual block, which indicated a block transition in both the mPFC and lPFC (Matsumoto, Matsumoto, Tanaka 2007). The cells with preblock-transition activities were intermingled with the cells responding to the first appearance of a visual stimulus in the mPFC and lPFC (Figs 7 and 8). This mixed distribution might help the former cells learn the probability of the block transition by locally gathering signals from the latter cells. The mixed distribution is also consistent with the idea of some theories that uncertainty about the value of a particular variable is bound to the representation of the value of that variable (Dayan et al. 1995; Hinton and Dayan 1996; Friston 2009; Friston and Kiebel 2009), as discussed above for the relationship between the cells with prefeedback activities in C1/E1 and feedback-responsive cells.

While the cells with prefeedback activities in C1/E1 were intermingled with the cells with preblock-transition activities in the mPFC and lPFC, the overlap of the two types of activities in the same cells was small in both areas. Moreover, the prefeedback activities and preblock-transition activities were negatively correlated across cells. This suggests that the prefrontal neural circuitry independently processed the uncertainty about the correctness of the executed action and the uncertainty about the transition from an action-learning block to a visual block. The uncertainty about the correctness of the executed action might correspond to the rule uncertainty in the framework of Bach and Dolan (Bach and Dolan 2012), because the rule for each action-learning block was uncertain, particularly in C1/E1, in terms of which action (left or right lever press) causes the correct feedback (Supplementary Fig. 3). The uncertainty about the transition from an action-learning block to a visual block might correspond to the state uncertainty (Bach and Dolan 2012), because it is uncertain after three or more consecutive correct trials in the action-learning block, in terms of which trial state the task is going to, the state of the action-learning block, or the state of the visual block (Supplementary Fig. 3). Thus, these findings support the view that distinct neural circuits represent different types of uncertainty, as discussed by Bach and Dolan (Bach and Dolan 2012).

In the present study, we did not find a clear difference between the mPFC and lPFC in terms of the frequency or localization of the activities representing either type of uncertainty. This is understandable because the signals in the mPFC that specify the type and strength of cognitive control are sent from the mPFC to the lPFC to implement the control (Kerns et al. 2004; Shenhav et al. 2013; Cavanagh and Frank 2014; Botvinick and Braver 2015; Moore and Zirnsak 2017; Badre and Nee 2018). The later onset of the prefeedback activities in the lPFC is consistent with the idea that the signals originate in the mPFC, sent from the mPFC to the lPFC, and implemented for control in the lPFC. As for the onset of preblock-transition activities, we did not find a significant difference between the mPFC and lPFC. This might be due to the relatively compact time course of the activities. Thus, the present results provide only a limited amount of information regarding the functional differences between the mPFC and lPFC.

We previously reported that activities of cells in the mPFC and lPFC represented specific action-outcome combinations in a task in which the monkey selected an action on the basis of action-outcome contingency (Matsumoto et al. 2003). We did not find cell activities representing specific action-outcome combinations in the present study. This may be due to differences between the tasks. Particularly, there was a visual cue indicating the combination of action to execute and its outcome and the cue changed every trial in Matsumoto et al. (2003). In the task used in the present study, there was no cue and the correct action and the outcome (visual stimulus) associated with it were fixed within a block.

It has been found that midbrain dopamine cells, besides the prominent transient activities reflecting the prediction errors brought by stimuli or reward, showed a slower time course of activity before the onset of reward, reflecting the uncertainty of reward (Fiorillo et al. 2003; Aron et al. 2004; Preuschoff et al. 2006; Schultz 2006). Furthermore, previous studies (Yu and Dayan 2005; Payzan-LeNestour et al. 2013) have suggested that cells in cholinergic and noradrenergic nuclei signal the expected uncertainty and unexpected uncertainty, respectively. Because the mPFC not only receives afferents from the dopaminergic, cholinergic, and noradrenergic cells (Porrino and Goldman-Rakic 1982; Mesulam et al. 1986; Lewis et al. 1988; Lewis and Morrison 1989; Ghashghaei and Barbas 2001) but also projects to them directly and indirectly (Arnsten and Goldman-Rakic 1984; Chiba et al. 2001; Frankle et al. 2006; Haber and Behrens 2014), the uncertainty representation in the mPFC may be formed through reciprocal interaction with the neuromodulatory systems. In the medial basal forebrain, which includes cholinergic cells, Monosov et al. (2015) found cells showing gradually increasing activity toward the uncertain reward, although the recorded cells themselves were likely noncholinergic. Regarding the relation to noradrenergic cells, it has been suggested that the representation of uncertainty in the mPFC may put the locus coeruleus into a tonic mode, which in turn drives exploration (Aston-Jones and Cohen 2005; Payzan-LeNestour et al. 2013; Muller et al. 2019).

An fMRI study reported that compared with normal subjects, patients with schizophrenia overestimated the context change when they were asked to estimate the change in the context that stochastically determined the numbers presented sequentially (Kaplan et al. 2016). The functional connectivity in the PFC network at the time of context change judgment was weaker in the patients than in normal controls, and the authors suggested that the impairment of PFC functional connectivity underlaid the heightened inference of context change in the patients. They also found that the patients showed stronger functional connectivity between the PFC and midbrain when feedback about the correctness of their context change judgment was given. The authors suggested that the PFC adjusts the activities of dopaminergic neurons, which are involved in the evaluation of context change judgment, and that the adjustment was abnormal in the patients. The PFC neuronal activities reported in the present study may contribute to the adjustment of dopaminergic activities, which are important for efficient learning of action values in the action-learning blocks and of stimulus values in the visual blocks (Tobler et al. 2005; Diederen and Schultz 2015).

We found that the different groups of cells in the mPFC and lPFC represented two different types of uncertainty. Moreover, the strengths of both activities quantitatively represented the degree of the respective uncertainty. These findings suggest that different circuits represent different types of uncertainties.

## Authors’ Contributions

MM, KT, and KM designed the research; MM and KM performed the research; MM, HA, KT, and KM analyzed the data; MM, KT, and KM wrote the paper; and KM supervised the study.

## Supplementary Material

Supplementary_information-CCC-Matsumoto_b_tgac002

## References

[ref1] Arnsten AF, Goldman-Rakic PS. Selective prefrontal cortical projections to the region of the locus coeruleus and raphe nuclei in the rhesus monkey. Brain Res. 1984:306:9–18. 6466989 10.1016/0006-8993(84)90351-2

[ref2] Aron AR, Shohamy D, Clark J, Myers C, Gluck MA, Poldrack RA. Human midbrain sensitivity to cognitive feedback and uncertainty during classification learning. J Neurophysiol. 2004:92:1144–1152. 15014103 10.1152/jn.01209.2003

[ref3] Aston-Jones G, Cohen JD. An integrative theory of locus coeruleus-norepinephrine function: adaptive gain and optimal performance. Annu Rev Neurosci. 2005:28:403–450. 16022602 10.1146/annurev.neuro.28.061604.135709

[ref4] Bach DR, Dolan RJ. Knowing how much you don't know: a neural organization of uncertainty estimates. Nat Rev Neurosci. 2012:13:572–586. 22781958 10.1038/nrn3289

[ref5] Badre D, Nee DE. Frontal cortex and the hierarchical control of behavior. Trends Cogn Sci. 2018:22:170–188. 29229206 10.1016/j.tics.2017.11.005 PMC5841250

[ref6] Barbas H, Pandya DN. Architecture and intrinsic connections of the prefrontal cortex in the rhesus monkey. J Comp Neurol. 1989:286:353–375. 2768563 10.1002/cne.902860306

[ref7] Behrens TE, Woolrich MW, Walton ME, Rushworth MF. Learning the value of information in an uncertain world. Nat Neurosci. 2007:10:1214–1221. 17676057 10.1038/nn1954

[ref8] Botvinick M, Braver T. Motivation and cognitive control: from behavior to neural mechanism. Annu Rev Psychol. 2015:66:83–113. 25251491 10.1146/annurev-psych-010814-015044

[ref9] Cavanagh JF, Frank MJ. Frontal theta as a mechanism for cognitive control. Trends Cogn Sci. 2014:18:414–421. 24835663 10.1016/j.tics.2014.04.012 PMC4112145

[ref10] Chiba T, Kayahara T, Nakano K. Efferent projections of infralimbic and prelimbic areas of the medial prefrontal cortex in the Japanese monkey, Macaca fuscata. Brain Res. 2001:888:83–101. 11146055 10.1016/s0006-8993(00)03013-4

[ref11] Christopoulos GI, Tobler PN, Bossaerts P, Dolan RJ, Schultz W. Neural correlates of value, risk, and risk aversion contributing to decision making under risk. J Neurosci. 2009:29:12574–12583. 19812332 10.1523/JNEUROSCI.2614-09.2009 PMC2794196

[ref12] Corbetta M, Miezin FM, Dobmeyer S, Shulman GL, Petersen SE. Selective and divided attention during visual discriminations of shape, color, and speed: functional anatomy by positron emission tomography. J Neurosci. 1991:11:2383–2402. 1869921 10.1523/JNEUROSCI.11-08-02383.1991 PMC6575512

[ref13] Critchley HD, Mathias CJ, Dolan RJ. Neural activity in the human brain relating to uncertainty and arousal during anticipation. Neuron. 2001:29:537–545. 11239442 10.1016/s0896-6273(01)00225-2

[ref14] Dayan P, Hinton GE, Neal RM, Zemel RS. The Helmholtz machine. Neural Comput. 1995:7:889–904. 7584891 10.1162/neco.1995.7.5.889

[ref15] Diederen KM, Schultz W. Scaling prediction errors to reward variability benefits error-driven learning in humans. J Neurophysiol. 2015:114:1628–1640. 26180123 10.1152/jn.00483.2015 PMC4563025

[ref16] van Doorn J, Ly A, Marsman M, Wagenmakers E-J. Bayesian rank-based hypothesis testing for the rank sum test, the signed rank test, and Spearman'sρ. J Appl Stat. 2020:47:2984–3006. 10.1080/02664763.2019.1709053 PMC9041780 35707708

[ref17] Fiorillo CD, Tobler PN, Schultz W. Discrete coding of reward probability and uncertainty by dopamine neurons. Science. 2003:299:1898–1902. 12649484 10.1126/science.1077349

[ref18] Frankle WG, Laruelle M, Haber SN. Prefrontal cortical projections to the midbrain in primates: evidence for a sparse connection. Neuropsychopharmacology. 2006:31:1627–1636. 16395309 10.1038/sj.npp.1300990

[ref19] Freedman D, Diaconis P. On the histogram as a density estimator:L2 theory. Z Wahrscheinlichkeitstheorie verw Gebiete. 1981:57:453–476.

[ref20] Friston K . The free-energy principle: a rough guide to the brain? Trends Cogn Sci. 2009:13:293–301. 19559644 10.1016/j.tics.2009.04.005

[ref21] Friston K, Kiebel S. Predictive coding under the free-energy principle. Philos Trans R Soc Lond Ser B Biol Sci. 2009:364:1211–1221. 19528002 10.1098/rstb.2008.0300 PMC2666703

[ref22] Ghashghaei HT, Barbas H. Neural interaction between the basal forebrain and functionally distinct prefrontal cortices in the rhesus monkey. Neuroscience. 2001:103:593–614. 11274781 10.1016/s0306-4522(00)00585-6

[ref23] Haber SN, Behrens TE. The neural network underlying incentive-based learning: implications for interpreting circuit disruptions in psychiatric disorders. Neuron. 2014:83:1019–1039. 25189208 10.1016/j.neuron.2014.08.031 PMC4255982

[ref24] Hinton GE, Dayan P. Varieties of Helmholtz machine. Neural Netw. 1996:9:1385–1403. 12662541 10.1016/s0893-6080(96)00009-3

[ref25] Ito S, Stuphorn V, Brown JW, Schall JD. Performance monitoring by the anterior cingulate cortex during saccade countermanding. Science. 2003:302:120–122. 14526085 10.1126/science.1087847

[ref26] Kaplan CM, Saha D, Molina JL, Hockeimer WD, Postell EM, Apud JA, Weinberger DR, Tan HY. Estimating changing contexts in schizophrenia. Brain. 2016:139:2082–2095. 27217338 10.1093/brain/aww095 PMC4939701

[ref27] Keri S, Decety J, Roland PE, Gulyas B. Feature uncertainty activates anterior cingulate cortex. Hum Brain Mapp. 2004:21:26–33. 14689507 10.1002/hbm.10150 PMC6871971

[ref28] Kerns JG, Cohen JD, MacDonald AW 3rd, Cho RY, Stenger VA, Carter CS. Anterior cingulate conflict monitoring and adjustments in control. Science. 2004:303:1023–1026. 14963333 10.1126/science.1089910

[ref29] Keysers C, Gazzola V, Wagenmakers EJ. Using Bayes factor hypothesis testing in neuroscience to establish evidence of absence. Nat Neurosci. 2020:23:788–799. 32601411 10.1038/s41593-020-0660-4 PMC7610527

[ref30] Koblinger A, Fiser J, Lengyel M. Representations of uncertainty: where art thou? Curr Opin Behav Sci. 2021:38:150–162. 34026948 10.1016/j.cobeha.2021.03.009 PMC8121756

[ref31] Lewis DA, Morrison JH. Noradrenergic innervation of monkey prefrontal cortex: a dopamine-beta-hydroxylase immunohistochemical study. J Comp Neurol. 1989:282:317–330. 2715385 10.1002/cne.902820302

[ref32] Lewis DA, Foote SL, Goldstein M, Morrison JH. The dopaminergic innervation of monkey prefrontal cortex: a tyrosine hydroxylase immunohistochemical study. Brain Res. 1988:449:225–243. 2899447 10.1016/0006-8993(88)91040-2

[ref33] Ma WJ, Jazayeri M. Neural coding of uncertainty and probability. Annu Rev Neurosci. 2014:37:205–220. 25032495 10.1146/annurev-neuro-071013-014017

[ref34] Matsumoto K, Suzuki W, Tanaka K. Neuronal correlates of goal-based motor selection in the prefrontal cortex. Science. 2003:301:229–232. 12855813 10.1126/science.1084204

[ref35] Matsumoto M, Matsumoto K, Abe H, Tanaka K. Medial prefrontal cell activity signaling prediction errors of action values. Nat Neurosci. 2007:10:647–656. 17450137 10.1038/nn1890

[ref36] Matsumoto M, Matsumoto K, Tanaka K. Effects of novelty on activity of lateral and medial prefrontal neurons. Neurosci Res. 2007:57:268–276. 17137664 10.1016/j.neures.2006.10.017

[ref37] McGuire JT, Nassar MR, Gold JI, Kable JW. Functionally dissociable influences on learning rate in a dynamic environment. Neuron. 2014:84:870–881. 25459409 10.1016/j.neuron.2014.10.013 PMC4437663

[ref38] Mesulam MM, Mufson EJ, Wainer BH. Three-dimensional representation and cortical projection topography of the nucleus basalis (Ch4) in the macaque: concurrent demonstration of choline acetyltransferase and retrograde transport with a stabilized tetramethylbenzidine method for horseradish peroxidase. Brain Res. 1986:367:301–308. 3516304 10.1016/0006-8993(86)91607-0

[ref39] Monosov IE . Anterior cingulate is a source of valence-specific information about value and uncertainty. Nat Commun. 2017:8:134. 28747623 10.1038/s41467-017-00072-y PMC5529456

[ref40] Monosov IE, Leopold DA, Hikosaka O. Neurons in the primate medial basal forebrain signal combined information about reward uncertainty, value, and punishment anticipation. J Neurosci. 2015:35:7443–7459. 25972172 10.1523/JNEUROSCI.0051-15.2015 PMC4429151

[ref41] Moore T, Zirnsak M. Neural mechanisms of selective visual attention. Annu Rev Psychol. 2017:68:47–72. 28051934 10.1146/annurev-psych-122414-033400

[ref42] Muller TH, Mars RB, Behrens TE, O'Reilly JX. Control of entropy in neural models of environmental state. elife. 2019:8:e39404. doi: 10.7554/eLife.39404. PMC6395063 30816090

[ref43] Payzan-LeNestour E, Dunne S, Bossaerts P, O'Doherty JP. The neural representation of unexpected uncertainty during value-based decision making. Neuron. 2013:79:191–201. 23849203 10.1016/j.neuron.2013.04.037 PMC4885745

[ref44] Porrino LJ, Goldman-Rakic PS. Brainstem innervation of prefrontal and anterior cingulate cortex in the rhesus monkey revealed by retrograde transport of HRP. J Comp Neurol. 1982:205:63–76. 6121826 10.1002/cne.902050107

[ref45] Preuschoff K, Bossaerts P, Quartz SR. Neural differentiation of expected reward and risk in human subcortical structures. Neuron. 2006:51:381–390. 16880132 10.1016/j.neuron.2006.06.024

[ref46] Quilodran R, Rothe M, Procyk E. Behavioral shifts and action valuation in the anterior cingulate cortex. Neuron. 2008:57:314–325. 18215627 10.1016/j.neuron.2007.11.031

[ref47] Rushworth MF, Behrens TE. Choice, uncertainty and value in prefrontal and cingulate cortex. Nat Neurosci. 2008:11:389–397. 18368045 10.1038/nn2066

[ref48] Schultz W . Behavioral theories and the neurophysiology of reward. Annu Rev Psychol. 2006:57:87–115. 16318590 10.1146/annurev.psych.56.091103.070229

[ref49] Seo H, Lee D. Temporal filtering of reward signals in the dorsal anterior cingulate cortex during a mixed-strategy game. J Neurosci. 2007:27:8366–8377. 17670983 10.1523/JNEUROSCI.2369-07.2007 PMC2413179

[ref50] Shenhav A, Botvinick MM, Cohen JD. The expected value of control: an integrative theory of anterior cingulate cortex function. Neuron. 2013:79:217–240. 23889930 10.1016/j.neuron.2013.07.007 PMC3767969

[ref51] Soltani A, Izquierdo A. Adaptive learning under expected and unexpected uncertainty. Nat Rev Neurosci. 2019:20:635–644. 31147631 10.1038/s41583-019-0180-y PMC6752962

[ref52] Tobler PN, Fiorillo CD, Schultz W. Adaptive coding of reward value by dopamine neurons. Science. 2005:307:1642–1645. 15761155 10.1126/science.1105370

[ref53] Walker AE . A cytoarchitectural study of the prefrontal area of the macaque monkey. J Comp Neurol. 1940:73:59–86.

[ref54] Watkins C, Dayan P. Q-learning. Mach Learn. 1992:8:279–292.

[ref55] Yu AJ, Dayan P. Uncertainty, neuromodulation, and attention. Neuron. 2005:46:681–692. 15944135 10.1016/j.neuron.2005.04.026

