# Supplementary material for: Different types of uncertainty distinguished by monkey prefrontal neurons
Source: Cereb Cortex Commun. 2022 Jan 11;3(1):tgac002. doi: 10.1093/texcom/tgac002 (PMC8842276; doi:10.1093/texcom/tgac002)
Supplement: Supplementary_information-CCC-Matsumoto_b_tgac002 [file supplementary_information-ccc-matsumoto_b_tgac002.pdf]

Supplementary materials for:

Different Types of Uncertainty Distinguished by Monkey Prefrontal Neurons

Madoka Matsumoto[1,2,3,4*], Hiroshi Abe[3,4], Keiji Tanaka[4] and Kenji Matsumoto[2,4]

[1]Department of Preventive Intervention for Psychiatric Disorders, National Institute of Mental Health, National Center of Neurology and Psychiatry, 4-1-1 Ogawahigashi-cho, Kodaira, Tokyo 187-8553, Japan.

[2]Tamagawa University Brain Science Institute, 6-1-1 Tamagawa-gakuen, Machida, Tokyo 194-8610, Japan.

[3]Laboratory for Molecular Analysis of Higher Brain Function, Center for Brain Science, RIKEN, 2-1 Hirosawa, Wako, Saitama 351-0198, Japan.

[4]Laboratory for Cognitive Brain Mapping, Center for Brain Science, RIKEN, 2-1 Hirosawa, Wako, Saitama 351-0198, Japan.

Supplementary Data

Supplementary Table 1

Supplementary Table 2

Supplementary Figure 1

Supplementary Figure 2

Context		After C1/eC1	After C2/eC2	After C3/eC3	After C4/eC4	After V1	After V2
Probability	Monkey 1	0	0	0.35	0.57	1	1
	Monkey 2	0	0	0.36	0.59	1	1
Entropy	Monkey 1	0	0	0.93	0.99	0	0
	Monkey 2	0	0	0.94	0.98	0	0

Supplementary Table 1. Uncertainty about transition from an action-learning block to a visual block. Probabilities of the block transition (upper) and entropy calculated from them (lower) are shown for trials in different contexts.

Monkey 1	First trial		After c		After cc		After e		After ec		After ecc	
	C	E	C	E	C	E	C	E	C	E	C	E
actual $P_{correct}$, P_{error}	0.561	0.439	0.990	0.010	0.998	0.002	0.909	0.091	0.996	0.004	0.998	0.002
$\boldsymbol{r_{pos}}, \boldsymbol{r_{neg}}$	1	-0.435	1	-0.435	1	-0.435	1	-0.435	1	-0.435	1	-0.435
$\bar{\boldsymbol{r}}$	0.370		0.985		0.997		0.870		0.994		0.998	
$SD_{feedback}$	0.712		0.145		0.060		0.412		0.089		0.057	
estimated $P_{correct}$, P_{error}	0.5	0.5	0.994	0.006	0.996	0.004	0.903	0.097	0.996	0.004	0.996	0.004
$\delta Q_{correct}$, δQ_{error}	1	-0.435	0.075	0.439	0.006	0.505	0.620	-0.033	0.047	0.509	0.003	0.550
$\lvert\delta Q_{correct}\rvert$, $\lvert\delta Q_{error}\rvert$	1	0.435	0.075	0.439	0.006	0.505	0.620	0.033	0.047	0.509	0.003	0.550
EV_{PE}	0.717		0.077		0.008		0.563		0.049		0.005	

Monkey 2	First trial		After c		After cc		After e		After ec		After ecc	
	C	E	C	E	C	E	C	E	C	E	C	E
actual $P_{correct}$, P_{error}	0.501	0.499	0.987	0.013	0.987	0.013	0.933	0.067	0.982	0.018	0.993	0.007
$\boldsymbol{r_{pos}}, \boldsymbol{r_{neg}}$	1	-0.449	1	-0.449	1	-0.449	1	-0.449	1	-0.449	1	-0.449
$\bar{\boldsymbol{r}}$	0.278		0.981		0.982		0.903		0.974		0.990	
$SD_{feedback}$	0.724		0.167		0.162		0.362		0.193		0.117	
estimated $P_{correct}$, P_{error}	0.5	0.5	0.987	0.013	0.987	0.013	0.876	0.124	0.987	0.013	0.987	0.013
$\delta Q_{correct}$, δQ_{error}	1	-0.449	0.002	0.547	0.000	0.550	0.552	-0.001	0.001	0.549	0.000	0.551
$\lvert\delta Q_{correct}\rvert$, $\lvert\delta Q_{error}\rvert$	1	0.449	0.002	0.547	0.000	0.550	0.552	0.001	0.001	0.549	0.000	0.551
EV_{PE}	0.7246		0.009		0.007		0.484		0.008		0.007	

Supplementary Table 2. Actual and estimated behavioral values used to calculate $SD_{feedback}$ and EV_{PE}.

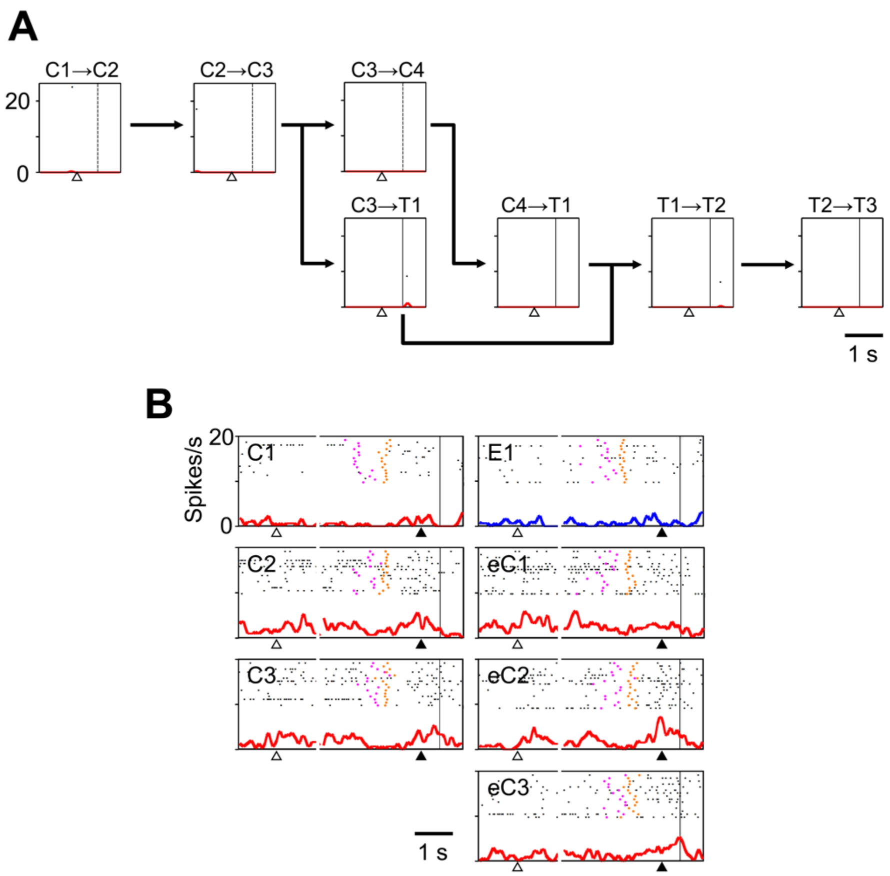

Supplementary Figure 1. (**A**) Absence of pre-block-transition activity in a cell with significant pre-feedback activities in C1/E1 recorded in the mPFC (the same cell as **Fig. 2A**). The cell did not show activity during the fixation period after C3, eC3, C4, or eC4. Abbreviations are the same as those in **Figure 5B**. (**B**) Absence of pre-feedback activity in a cell with significant pre-block-transition activities recorded in the mPFC (the same cell as **Fig. 5B**). The cell did not show any activity during the pre-feedback period in C1, E1, and eC1. Abbreviations are the same as those in **Figures 2A,B**.

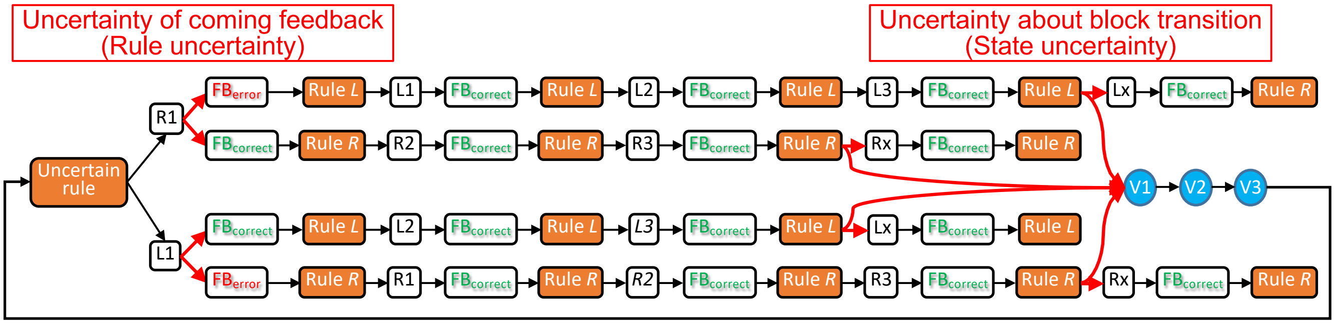

Supplementary Figure 2. Detailed schema of the two types of uncertainty in the task. The uncertainty of the coming feedback is the uncertainty about the rules between "Right choice causes correct feedback" (Rule *L*) and "Left choice causes correct feedback" (Rule *R*) regardless of the executed action. This type of uncertainty corresponds to the rule uncertainty in the framework of Bach and Dolan (2012). The uncertainty about block transition is the uncertainty about whether the current state is still in the action-learning block or is moving to the visual block. This type of uncertainty corresponds to state uncertainty in the framework of Bach and Dolan (2012). R1, R2, R3, and Rx indicate the first, second, third, and the following x-th right choices in the action-learning block, respectively. L1, L2, L3, and Lx indicate the first, second, third, and x-th left choices in the action-learning block, respectively. $FB_{correct}$ and FB_{error} indicate visual feedback for the correct choice and that for erroneous choices.
